# Clinical prediction models using machine learning in oncology: challenges and recommendations

**DOI:** 10.1136/bmjonc-2025-000914

**Published:** 2025-10-07

**Authors:** Gary S Collins, Mae Chester-Jones, Stephen Gerry, Jie Ma, Joao Matos, Jyoti Sehjal, Biruk Tsegaye, Paula Dhiman

**Affiliations:** 1 Department of Applied Health Sciences, University of Birmingham, Birmingham, UK; 2 University of Oxford, Oxford, UK

**Keywords:** Epidemiology

## Abstract

Clinical prediction models are widely developed in the field of oncology, providing individualised risk estimates to aid diagnosis and prognosis. Machine learning methods are increasingly being used to develop prediction models, yet many suffer from methodological flaws limiting clinical implementation. This review outlines key considerations for developing robust, equitable prediction models in cancer care. Critical steps include systematic review of existing models, protocol development, registration, end-user engagement, sample size calculations and ensuring data representativeness across target populations. Technical challenges encompass handling missing data, addressing fairness across demographic groups and managing complex data structures, including censored observations, competing risks or clustering effects. Comprehensive internal and external evaluation requires assessment of both statistical performance (discrimination and calibration) and clinical utility. Implementation barriers include limited stakeholder engagement, insufficient clinical utility evidence, a lack of consideration of workflow integration and the absence of post-deployment monitoring plans. Despite significant potential for personalising cancer care, most prediction models remain unimplemented due to these methodological and translational challenges. Addressing these considerations from study design through post implementation monitoring is essential for developing trustworthy tools that bridge the gap between model development and clinical practice in oncology.

## Introduction

 Prediction models have long been used to guide clinical decision making and fall broadly into one of two categories: diagnostic or prognostic.^1^ Diagnostic prediction models estimate the probability of a specific condition (typically a disease) being present, while a prognostic prediction model estimates the probability of developing a specific health outcome over a specific time period. One of the primary benefits of multivariable prediction models is their superior predictive accuracy compared with simpler risk classification systems or single prognostic factors. By incorporating multiple predictors simultaneously, these models use all available relevant information to generate more precise individualised risk estimates. This approach is particularly important in oncology, where disease progression and treatment response are influenced by numerous interacting factors.

Cancer care is inherently prediction-driven, with countless clinical decisions, from screening and prevention to treatment selection and palliative care transitions, all fundamentally dependent on assessing the likelihood of future events.^2^ Traditional approaches to prediction in oncology have heavily relied on categorical risk classifications such as cancer staging systems, which assign patients to broad risk groups based on limited clinical variables. However, these approaches are limited in their ability to capture the complex and multifactorial nature of cancer progression. Prediction modelling offers a more nuanced, data-driven approach to individualised risk estimation that better addresses the inherent complexities of cancer care.

Many prediction models have been developed for various cancers, including kidney,^3^ breast,^4^ oral,^5^ gastric,^6^ colorectal,^7^ endometrial,^8^ pancreas,^9^ melanoma,^10^ head and neck,^11^ lung,^12^ oesophageal^13^ and cervical.^14^ Well-known examples, within the field of oncology, include models such as the Gail model to estimate the 5-year and lifetime breast cancer risk,^15^ the PREDICT tool for estimating survival following surgery for invasive breast cancer,^16^ and the Prostate Biopsy Collaborative Group model for estimating the risk of high-grade prostate cancer on biopsy.^17^

Evidence demonstrates that multivariable clinical prediction models consistently outperform simpler risk classification methods across various cancer types.^18^ For example, greater accuracy has been found using models than clinical staging alone for predicting disease-specific survival in gastric cancer,^19^ or better patient outcomes than pathological stage.^20^ This improved accuracy translates directly to better clinical decision-making by providing more reliable information about individual patient risks and potential benefits of different treatment options. Another advantage of prediction models is their ability to incorporate novel predictors such as genomic data, imaging features and other emerging biomarkers. As our understanding of cancer biology advances, prediction models provide a structured framework for evaluating and integrating new predictors. The flexible nature of these models allows for regular refinement as new evidence emerges. When a promising new biomarker is identified, it can be evaluated for its incremental predictive value within existing models.^21^ This process, in principle, ensures that clinical prediction incorporates the most current scientific knowledge while maintaining statistical rigour.

Clinical prediction models have traditionally been developed using regression approaches. For example, logistic regression is typically used to predict binary outcomes (such as the presence or absence of cancer) and the Cox proportional hazards model for time-to-event outcomes (such as survival or recurrence).^22^ These statistical methods integrate multiple predictors to generate a mathematical formula that calculates individualised risk estimates. More recently, machine learning approaches have expanded the modeller’s toolkit for developing prediction models, offering potential advantages in handling complex, multimodal and non-linear relationships between predictors and outcomes.^23^ These techniques range from decision trees and random forests to neural networks and deep learning methods, each with specific strengths, complexities and limitations for clinical prediction.

Despite their potential benefits to improve care and outcomes for oncology patients, the development, evaluation and implementation of clinical prediction models face several challenges that can limit their implementation. There is overwhelming evidence that many prediction models, including those involving machine learning, suffer from poor design and methods,^24^,^26^ incomplete reporting,^27 28^ are at high risk of bias^29 30^ and exhibit overinterpretation.^31 32^ In this article, we outline some key steps researchers should consider when developing or validating a clinical prediction model (table 1).

**Table 1:** Key considerations when developing and evaluating the performance of a clinical prediction model: challenges and recommendations

Step	Challenges	Recommendations
Clinical purpose and end-user involvement	Lack of early and meaningful stakeholder engagement may lead to models that are clinically irrelevant or misaligned with real-world workflows. Duplication of effort and model proliferation without added value.^80^	Engage end-users (eg, healthcare professionals, patients) early to align the model with a clinical need, the decision the model is intended to support, target population and setting. Avoid redundancy by reviewing and comparing (head-to-head) existing models. Consider updating any existing model before developing a new model.
Protocol and registration	Absence of protocol registration reduces transparency, increasing the risk of selective reporting and methodological inconsistency.	Register the study, for example, on clinicaltrials.gov, and prepare a study protocol, describing all aspects of the model development and evaluation, and make this publicly available.^42^
Study design and data	Retrospective, repurposed or non-representative data may compromise the validity and generalisability of the model.	Ideally, use designs with prospective data collection (cross-sectional for diagnostic, longitudinal for prognostic models). Data should be representative of the target population and setting of intended use.
Sample size	Calculating the sample size is required for developing clinical prediction models,^44^ but this is rarely done in the literature.^26^ Small sample sizes lead to unstable models (*development*) and unreliable performance metrics (*evaluation*).	*Model development*: Ensure the sample size is sufficiently large enough to develop a model so that overfitting is minimised.^44^ *Model evaluation*: Ensure the sample size is sufficiently large enough to reliably and precisely estimate model performance.^45^
Missing data	Inadequate handling of missing data may introduce bias, reduce model generalisability and hinder clinical uptake.	Avoid excluding individuals with incomplete data; instead, explore methods for imputing those missing values. Carefully consider whether missing data will be when the model is used in practice and plan how to handle those missing values during both model development and evaluation.
Fairness	Biases in data or analysis can lead to unfair predictions, potentially creating or worsening existing health inequities.	Ensure aspects of fairness are embedded and addressed, for example, through diverse stakeholder involvement, design, data curation, analysis and reporting.^64^
Model complexities	Ignoring data-specific complexities may produce misleading inferences and limit clinical utility.	Ensure complexities in the data are appropriately analysed, for example, censored observations, competing risks,^67^ clustering^81^ and recurrent events.^82^
Model evaluation	Reliance on internal validation alone (using model development data) may mask poor generalisability. Limited external validation reduces trust and real-world applicability.	*Internal validation*: use bootstrapping or cross-validation. *Internal-external validation*: when possible (eg, large, clustered data), explore heterogeneity in model performance, where a prediction model is iteratively developed on data from multiple subsets (eg, hospitals, geographic locations) and validated on the remaining excluded subsets. *External validation*: evaluate the model in new data to assess generalisability.^74^
Model stability	The larger the instability in model outputs, the greater the threat that the developed model has poor internal or external validity.	Consider stability checks during model development to ensure predictions are reproducible and trustworthy.^83^
Model performance	Selected performance metrics are not always proper or centred on patient outcomes, with relevance in clinical scenarios.	Assess model discrimination (eg, c-statistic), calibration (calibration plots) and clinical utility (net benefit).^75^
Translation and implementation barriers	Failure to account for implementation barriers (eg, interoperability, clinician burden, automation bias^84^) may limit real-world impact.	Consider how the model will be used in practice and how best to facilitate adoption.^85^
Post-deployment monitoring	Models need to be monitored after deployment to ensure they maintain their performance over time.	Include provisions for ongoing evaluation and refinement, consider statistical performance, practical utility and clinical impact.
Reporting	Incomplete or inconsistent reporting hinders trust, reproducibility, study comparison and critical appraisal of models.	Transparently report all aspects of the development and validation of the clinical prediction model by following the TRIPOD+AI reporting guideline.^78^

TRIPOD+AI, Transparent reporting of a multivariable prediction model for Individual Prognosis Or Diagnosis + Artificial Intelligence.

## Do we need yet another prediction model?

There are often many published models developed for the same purpose, for example, >900 models for supporting breast cancer decision-making (including >400 for predicting mortality and >200 for predicting recurrence),^33^ or the >100 prognostic models for predicting overall survival in patients with gastric cancer.^34^ Therefore, before developing a new model, consider conducting a (systematic) review of the literature to identify,^35 36^ critically appraise^37^ and, where appropriate, evaluate and update any existing and promising models.^38^ If and only if none of the existing models can be used in the target setting, consider developing a new model. The findings from carrying out a review of existing models can also help inform model development, that is, identifying commonly used predictors, or ensuring no flaws or critical shortcomings in existing models are repeated.

## End-user involvement

At the outset, end-user involvement, including clinicians, patients and the public, is critical in developing a clinical prediction model to ensure it is relevant, usable and trustworthy in real-world settings.^39^ Engaging clinicians helps clarify the clinical questions the model must address, informs the selection of meaningful predictors and guides how predictions will be integrated into clinical workflows, which is essential for implementation and impact.^40^

 Involving patients and the public ensures that models reflect lived experiences, prioritise outcomes that matter most to those affected and address concerns about data use, fairness and transparency.^41^ This collaborative approach also improves the accuracy, understandability and practical utility of prediction models, as end users can provide feedback on whether outputs are actionable and aligned with their needs. Ultimately, end-user involvement in determining whether a model is actually needed in a specific population and setting. Their involvement throughout model development enhances the likelihood that prediction tools will be effectively implemented, ethically sound and capable of improving patient care and decision-making.

## Protocol and registration

A study protocol is an essential document for any research project, detailing its purpose, design, data collection, analysis methods, patient and public involvement, data/code sharing, dissemination plans and overall organisation.^42^ For certain studies, like randomised controlled trials, protocols are mandatory for funding, ethics approval and publication. In the context of machine learning in healthcare, where prediction model studies are rapidly increasing and systematic reviews often highlight methodological flaws, protocols are vital for reducing research waste and improving study quality. Developing a protocol forces researchers to plan thoroughly, reducing future problems and enhancing transparency, reproducibility and research integrity. Publicly available protocols also allow readers to compare planned versus actual methods and results and facilitate peer review. We acknowledge, however, that in the case of machine learning or other adaptive modelling approaches, a protocol may necessarily specify areas of uncertainty about the final modelling strategies to be employed. Rather than undermining protocol registration, this openness provides readers with important context to assess the planned flexibility and the rigour of the process.

While there is currently no mandatory requirement to register machine learning-driven prediction model research, registering can reduce selective reporting and inflated performance claims, fostering greater trust. Platforms like ClinicalTrials.gov and the Open Science Framework allow registration of studies and depositing of study protocols, with options to embargo details until publication to prevent being scooped. Registration promotes higher standards and trustworthiness in prediction model research.

## Study design: sample size

Study design is crucial, as it guides data collection, the choice of analysis methods and validity of findings. In health research, where findings impact patient care, public health policies and resource allocation, flawed design can lead to misleading conclusions, wasted resources and even harm, if implemented into a workflow. Sample size is a critical design component of all studies and a key consideration in predictive Artificial Intelligence (AI) research. Yet, despite its importance, the data used to train and test predictive AI, even before any data splitting, are often too small and rarely justified.^43^

Sample size calculations are crucial and arguably one of the most important considerations for studies developing and validating clinical prediction models, as inadequate sample sizes can lead to overfitted models with poor performance when applied to new patients. For regression-based approaches, guidance on minimum sample size has been proposed for model development^44^ and validation,^45^ which have been implemented in R and Stata statistical software ‘pmsampsize’ and ‘pmvalsampsize’, respectively. However, for other machine learning methods, sample size requirements often differ substantially and are more difficult to determine.

Machine learning algorithms typically require larger datasets due to their ability to model complex, non-linear relationships and interactions, with requirements varying based on the specific algorithm (eg, random forests, neural networks, support vector machines).^46^ Unlike regression methods, many machine learning approaches lack established sample size formulas. Researchers will typically have to rely on simulation studies, learning curves^47^ or performance plateaus to gauge whether the sample size is adequate. Some other emerging methods include simulation or Bayesian-driven approaches that estimate minimum sample sizes by examining model performance, degradation and stability.^48^,^50^ However, this methodological gap highlights the need for further research to develop sample size calculation methods for prediction models using machine learning techniques that can align with established guidance available for regression-based prediction models.

## Study design: data representativeness

It is important that the data used to train a clinical prediction model is representative of the target population in whom the model is intended to be used. Representative data ensures that the model accounts for the demographic, clinical and socioeconomic diversity of the population, reducing biases that could lead to inaccurate predictions or inequitable health outcomes for underrepresented groups (eg, ethnic minorities). Without such representativeness, models risk perpetuating disparities, as they will likely fail to generalise to real-world settings. Using existing data which were collected for purposes other than developing a prediction model, out of mere convenience, needs careful consideration as it may not reflect contemporary target populations, have data quality issues or have not collected information on important predictors. Similarly, using data from a different geographic setting is important, as there is often substantial variation in patient demographics, diagnosis, care, treatment and health outcomes. For example, requiring a model for use in China but using training data from the USA (eg, SEER) needs careful consideration and justification.

Class imbalance refers to differences in the number of individuals with and without the outcome in a dataset. This imbalance is natural, expected and reflects the reality of clinical populations; it is not inherently problematic when developing a prediction model. Nevertheless, methods such as undersampling, oversampling or synthetic approaches like synthetic minority over-sampling technique (SMOTE) are often promoted as solutions to supposed concerns about imbalance. These methods artificially manipulate the data to balance outcome frequencies, but in doing so, they distort the underlying distribution and undermine the representativeness of the dataset. Far from being corrective, such approaches are fallacious and can harm the validity and generalisability of the resulting model. We caution against their use in clinical prediction model studies and refer the reader to further discussion of other problems created by ‘correcting’ for class imbalance.^51^

## Study design: data provenance

Detailing data provenance, the documented history of data sources, collection methods and handling procedures, serves as a crucial safeguard against research fraud. Detailed information on provenance enables verification of data authenticity, making fabricated or manipulated data more difficult to introduce undetected. Without detailed data provenance, the scientific community cannot effectively distinguish between genuine clinical findings and manufactured results, potentially leading to flawed models that could endanger patient safety if implemented in clinical practice. As high-profile cases of data fabrication continue to emerge in biomedical research, establishing transparent, auditable data lineage is an essential ethical requirement, ensuring that clinical prediction models are built on foundations of scientific integrity rather than fraudulent data.

## Missing data

A carefully designed study is crucial for minimising missing data. Careful planning of data collection procedures, clear definitions of variables and training of data collectors can reduce the occurrence of missing values. However, when missing data inevitably arise, they can introduce bias leading models with poor generalisability. Discarding individuals with any missing values and conducting a ‘complete-case’ analysis should generally be avoided. It can lead to bias under many missingness mechanisms, reduce sample size, discard important information increasing the risk of overfitting and affect the representativeness of the data, leading to a model with reduced or poor generalisability.

When missing data cannot be avoided, including using existing data which has missing values, it is therefore important to choose an appropriate method depending on the aim and setting. Importantly, the appropriate strategy depends on whether missing data will be permitted at model deployment and if so, how. Techniques such as multiple imputation (replacing missing values with multiple plausible estimates),^52^ regression imputation (using fitted models to predict missing values), reference values (eg, mean or median age-sex values) and missing indicator methods offer viable solutions depending on the model that will be used in practice.^53 54^ For some modelling approaches, handling missing data is built-in to the model training process (eg, XGBoost).

## Fairness

Fairness is increasingly emphasised in prediction model research, with recommendations for it to be considered at multiple stages of model development and evaluation. However, fairness considerations should begin at the stage of problem formulation. Researchers should question whether there are documented health inequities in disease incidence, screening, diagnosis, treatment or outcomes, leading to the essential first step of identifying clinically meaningful dimensions (ie, sensitive attributes) for fairness evaluation.

In oncology, where disease burden, access to care and clinical outcomes frequently differ across demographic and socioeconomic groups, addressing these disparities is critical to avoid exacerbating existing inequities. For example, prostate cancer incidence is higher in black patients.^55^ Further, incidence, pathological characteristics and clinical outcomes in breast cancer vary across geographical, racial and ethnic groups, disproportionately affecting both black and Hispanic populations, who also remain underrepresented in breast cancer clinical trials.^56^ Data representativeness is foundational. Models like the Khorana Score for estimating the risk of venous thromboembolism in patients with cancer (developed in a predominantly white population) may lack current representativeness.^57^ Genomics-driven cancer care faces similar challenges, with databases like The Cancer Genome Atlas overrepresenting European ancestry and underrepresenting Asian, African and Hispanic populations, risking poor generalisability.^58^

Predictor selection also impacts fairness. Excluding sensitive attributes (eg, race/ethnicity) can affect model performance, especially where disease burden differs.^59^ While ‘race-aware models’ might mitigate disparities,^60^ race/ethnicity is a complex social construct, inconsistently recorded,^61^ and intersects with other identities.^62^ Frameworks such as *GUIDE* (Guidance for Unbiased Predictive Information for Healthcare Decision-making and Equity) help navigate their use, balancing statistical validity and ethics.^59^

Fairness evaluation also requires more than aggregate metrics; population-level assessment can mask subgroup disparities. Stratified evaluation (eg, by cancer type, sex, age, socioeconomic status, race/ethnicity) and visual tools (calibration plots, decision curve analysis) are necessary to detect differential performance and guide mitigation (eg, subgroup-specific thresholds). See online supplemental 1 for an example.^63^

Finally, fairness cannot be achieved without diverse stakeholder engagement. Involving patients, clinicians, caregivers and policymakers is vital to ensure that models are aligned with the values and needs of the populations they are intended to serve.^64^

## Modelling complexities

Developing a clinical prediction model requires accounting for any complex data structures. Common complexities, particularly in the field of oncology, include censored observations, competing risks and clustering. A censored observation occurs when the exact event time (eg, death, progression) is unknown because the patient either exits the study before the event occurs or the event has not yet happened by the end of the observation period. If improperly handled (eg, excluding censored patients, or assuming they did not experience the event), it can lead to incorrect risk estimates and compromise clinical decision making. Common approaches for handling censoring when developing a prediction model include Cox proportional hazards regression, random survival forests or neural networks (eg, DeepSurv^65^).

Competing risks refer to events that preclude the event being predicted (eg, death may preclude disease recurrence) and require specific methods during model development to avoid overestimation of risks^66^ and for model validation.^67^ Recent examples implementing XGBoost and neural networks for breast cancer prognostication in the presence of competing risks can be found here.^68^,^70^

Clustering, for example, within hospital or geographical location, can also be important to address when developing a clinical prediction model since observations within the same cluster can be more similar (ie, in characteristics or care) to each other than to those in different clusters, resulting in cluster groups that are distinct yet still related.^71^ If clustering is ignored, models may fail to account for any within-group similarities and between-group differences, leading to biased risk estimation, reduced predictive performance and poor calibration. Accounting for any clustering can offer advantages in prediction model research by allowing exploration of heterogeneity in model performance across different clusters.^72^ By identifying the sources of this heterogeneity, it may facilitate better adaptation and tailoring of prediction models to specific clusters, ultimately creating models that are more generalisable and effective in diverse settings.

These complexities are not always isolated and can sometimes coexist; therefore, complex strategies are sometimes required to integrate the different components of the modelling approaches that deal with the complexities.

## Model evaluation

Model evaluation is a multistage process that examines a model’s predictive performance across different contexts and datasets. Initial assessment typically involves internal validation, using techniques such as bootstrapping or cross-validation to quantify overfitting and estimate performance within the development data. Data splitting is often done, but is not always wise or efficient.^73^ When working with large or clustered datasets, internal-external validation can provide further insight by iteratively training the model on data from certain subsets (eg, hospitals or regions) and validating it on excluded subsets. External validation, performed on entirely independent data, is essential for assessing the model’s generalisability to new populations and clinical environments.^74^

## Model performance

Comprehensive evaluation of prediction models should also address multiple dimensions of performance, including statistical validity, clinical utility and implementation feasibility. Statistical validation should assess both discrimination (the model’s ability to distinguish between patients who will and will not experience the outcome) and calibration (the agreement between predicted and observed outcomes). Common metrics include the area under the receiver operating characteristic curve (referred to as the area under the receiver operating characteristic curve or c-statistic) for discrimination and calibration curves for calibration.^75^ Clinical utility assessment should evaluate whether model-guided decisions lead to improved patient outcomes compared with standard care.^76^ This evaluation may involve impact studies that measure changes in clinical decisions, resource utilisation, patient satisfaction and health outcomes associated with model implementation. With the increase in developed AI models, other classification-based measures of model’s performance are often reported. However, these should be interpreted with caution as they are often dependent on arbitrary risk thresholds and may lead users to treatment decisions without full consideration of the predicted risk.

## Transparent reporting

Transparent reporting is fundamental in the development and evaluation of clinical prediction models, as it enables stakeholders, including clinicians, researchers, patients and policymakers, to critically appraise study design, assess risk of bias and determine the clinical usefulness of a model.^37^ Further, where the model itself is not reported, this impedes the model being evaluated and used in clinical practice. Incomplete or inaccurate reporting can conceal methodological flaws, hinder reproducibility and ultimately compromise patient safety if flawed models are implemented in clinical practice.

Transparent reporting fosters trust, enhances the credibility of research and supports the ethical obligation to communicate findings honestly and completely.^77^ Guidelines such as the Transparent Reporting of a multivariable prediction model for Individual Prognosis Or Diagnosis (TRIPOD+AI)^78^ provide structured recommendations to ensure comprehensive description of methods, data sources and results, thereby facilitating model evaluation, replication and responsible implementation in healthcare. Ultimately, transparent reporting is not optional but essential for advancing trustworthy, equitable and effective clinical prediction models.

## Translational and implementation barriers

Even methodologically sound prediction models face significant barriers to clinical implementation. The gap between model development and clinical use remains substantial, with few models successfully translated into routine practice.^79^ Several factors contribute to this translational gap, including:

1. Limited stakeholder engagement during model development.
2. Incomplete or poor statistical evaluation of model performance.
3. Insufficient evidence of clinical utility beyond statistical performance, with largely non-existent evaluation of prediction models as an intervention to guide treatment and improve patient outcomes.
4. No plan or intention to implement that model.
5. Lack of user-friendly interfaces that integrate seamlessly into clinical workflows.
6. Inadequate education and training for potential users.
7. Regulatory and implementation hurdles.
8. Lack of plans or framework for post implementation monitoring.

Addressing these barriers requires a more comprehensive approach to model development that considers implementation factors from the outset, during the design stage, rather than as an afterthought.

## Post-deployment monitoring

Prediction models require ongoing quality assurance and periodic updating to maintain their performance over time. Clinical practices, patient populations and treatment options evolve continuously, potentially affecting the relevance and accuracy of prediction models. Without systematic processes for monitoring and updating, models may become outdated and potentially misleading. The life cycle of a clinical prediction model should include provisions for ongoing evaluation, refinement and, when necessary, replacement. This quality assurance process should assess not only statistical performance but also practical utility and clinical impact. Regulatory and technical frameworks such as the Food and Drug Administration (FDA) Software as a Medical Device principles and operational approaches like Machine Learning Operations (MLOps) further support post-deployment surveillance, enabling structured monitoring, risk management and continuous improvement of prediction models in real-world practice.

## Conclusions

Prediction models, using statistics and increasingly machine learning methods, are often developed for personalised diagnostic and prognostic assessments to aid clinical decision making. However, there are numerous design and methodological challenges, including lack of end-user involvement, small sample sizes, missing data, model unfairness, failure to account for complexities like censoring or competing risks and weak evaluation of model performance. Therefore, despite their potential, many models remain unimplemented. Addressing these challenges will ensure robust, equitable prediction tools that enhance decision-making and outcomes in oncology, bridging the gap between innovation and clinical practice.

## Supplementary material

10.1136/bmjonc-2025-000914 online supplemental file 1

## Data Availability

No data are available.
