# Supplementary material for: Clinical prediction models using machine learning in oncology: challenges and recommendations
Source: BMJ Oncol. 2025 Oct 7;4(1):e000914. doi: 10.1136/bmjonc-2025-000914 (PMC12506039; doi:10.1136/bmjonc-2025-000914)
Supplement: online supplemental file 1 [file bmjonc-4-1-s001.docx]

| **Box 1. Limitations of the Khorana score in predicting venous thromboembolism (VTE) risk during chemotherapy (figures and captions taken from [61]; which is licenced under CC BY-NC-ND 4.0)**  The Khorana score is a clinical tool developed to predict VTE risk in ambulatory cancer patients receiving chemotherapy. It uses five baseline variables: cancer site, platelet count, haemoglobin level or use of erythropoiesis-stimulating agents, leukocyte count, and BMI, to stratify patients into low, intermediate, or high risk groups. Initial validation showed moderate accuracy (c-statistic 0.7). [86]. Despite its widespread use, the Khorana score has notable limitations. A Danish study of over 40,000 chemotherapy patients found it failed to effectively stratify VTE risk across all cancer types, especially when accounting for competing mortality risks. In cancers such as hepatobiliary/pancreatic, lung, and gynecologic, the score did not meaningfully differentiate risk, raising concerns about its clinical utility for these groups [63].  **Key challenges illustrated by this case:**   - It is critical to account for relevant modelling complexities, such as competing risk of mortality. Failure to do so can lead to biased estimates, such as overestimation of absolute risk. - Clinical prediction models must be rigorously evaluated for their intended clinical purpose. In this case, the model’s ability to stratify patients by six-month VTE risk with competing risk of mortality was the relevant clinical purpose (Figure A). - Fairness evaluation requires the identification of clinically meaningful dimensions for fairness analysis. Here, differences in model performance across cancer types were identified as relevant. Without a fairness evaluation across clinically relevant subgroups, these differences in model performance would have gone undetected (Figure B). - In the presence of model unfairness, a single decision threshold may not be appropriate for all subgroups. One potential approach to improve fairness is to apply subgroup-specific thresholds. - Data representativeness and a vigilant post-deployment monitoring to data shifts are essential to ensure model validity and fairness, particularly as population characteristics and clinical practices evolve over time and across settings.  \| 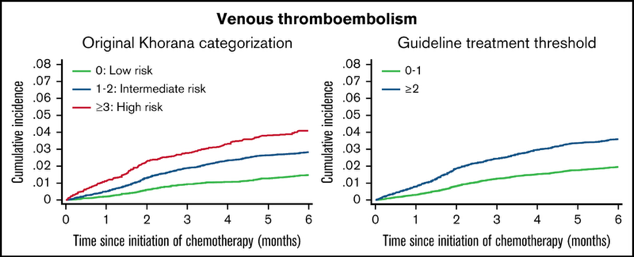**Figure A.** Six-month cumulative incidence of venous thromboembolism considering competing risk of death. Stratified by the original Khorana categorization (left). Stratified according to the current guideline-recommend score threshold for thromboprophylaxis (right).  **Interpretation:** Performance estimation at the population level may mask any (potential) differential model behaviour within that population. In these curves, Khorana score seems to perform an adequate risk stratification on the population level. Subgroup performance evaluation is essential for identifying groups where the model is underperforming. \| 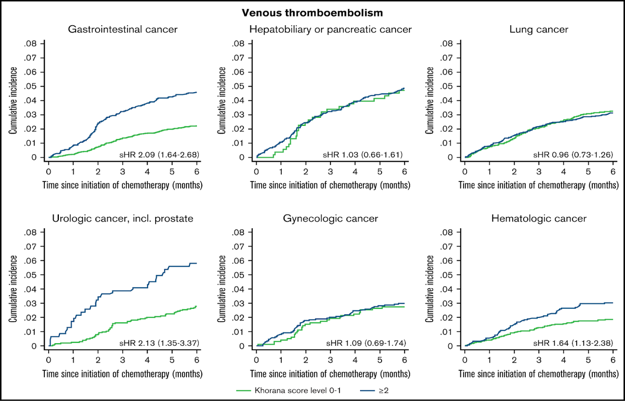  **Figure B.** Six-month cumulative incidence of venous thromboembolism considering competing risk of death and associated subdistribution hazard ratios [sHR (95% confidence interval)]. Stratified according to current guideline-recommended Khorana score threshold and by cancer type.  **Interpretation:** The risk stratification achieved by the Khorana score at the guideline-recommended threshold of 2 was not equally effective across cancer types, as indicated by the non-significant subdistribution hazard ratio estimates for the hepatobiliary/pancreatic, lung, and gynaecologic cancer subgroups. \| \| --- \| --- \| |
| --- | --- | --- |
